# Building a Sustainable Learning Health Care System for Pregnant and Lactating People: Interview Study Among Data Access Providers

**DOI:** 10.2196/47092

**Published:** 2024-02-08

**Authors:** Marieke J Hollestelle, Rieke van der Graaf, Miriam C J M Sturkenboom, Marianne Cunnington, Johannes J M van Delden

**Affiliations:** 1 Julius Center for Health Sciences and Primary Care University Medical Center Utrecht Utrecht Netherlands; 2 Bioethics & Health Humanities, University Medical Center Utrecht Utrecht Netherlands; 3 Data Science & Biostatistics, University Medical Center Utrecht Utrecht Netherlands; 4 Analysis Group Inc London United Kingdom

**Keywords:** ethics, learning health care systems, pregnancy, lactation, real-world data, governance, qualitative research

## Abstract

**Background:**

In many areas of health care, learning health care systems (LHSs) are seen as promising ways to accelerate research and outcomes for patients by reusing health and research data. For example, considering pregnant and lactating people, for whom there is still a poor evidence base for medication safety and efficacy, an LHS presents an interesting way forward. Combining unique data sources across Europe in an LHS could help clarify how medications affect pregnancy outcomes and lactation exposures. In general, a remaining challenge of data-intensive health research, which is at the core of an LHS, has been obtaining meaningful access to data. These unique data sources, also called data access providers (DAPs), are both public and private organizations and are important stakeholders in the development of a sustainable and ethically responsible LHS. Sustainability is often discussed as a challenge in LHS development. Moreover, DAPs are increasingly expected to move beyond regulatory compliance and are seen as moral agents tasked with upholding ethical principles, such as transparency, trustworthiness, responsibility, and community engagement.

**Objective:**

This study aims to explore the views of people working for DAPs who participate in a public-private partnership to build a sustainable and ethically responsible LHS.

**Methods:**

Using a qualitative interview design, we interviewed 14 people involved in the Innovative Medicines Initiative (IMI) ConcePTION (Continuum of Evidence from Pregnancy Exposures, Reproductive Toxicology and Breastfeeding to Improve Outcomes Now) project, a public-private collaboration with the goal of building an LHS for pregnant and lactating people. The pseudonymized transcripts were analyzed thematically.

**Results:**

A total of 3 themes were identified: opportunities and responsibilities, conditions for participation and commitment, and challenges for a knowledge-generating ecosystem. The respondents generally regarded the collaboration as an opportunity for various reasons beyond the primary goal of generating knowledge about medication safety during pregnancy and lactation. Respondents had different interpretations of responsibility in the context of data-intensive research in a public-private network. Respondents explained that resources (financial and other), scientific output, motivation, agreements collaboration with the pharmaceutical industry, trust, and transparency are important conditions for participating in and committing to the ConcePTION LHS. Respondents also discussed the challenges of an LHS, including the limitations to (real-world) data analyses and governance procedures.

**Conclusions:**

Our respondents were motivated by diverse opportunities to contribute to an LHS for pregnant and lactating people, primarily centered on advancing knowledge on medication safety. Although a shared responsibility for enabling real-world data analyses is acknowledged, their focus remains on their work and contribution to the project rather than on safeguarding ethical data handling. The results of our interviews underline the importance of a transparent governance structure, emphasizing the trust between DAPs and the public for the success and sustainability of an LHS.

## Introduction

### Background

In many areas of health care, learning health care systems (LHSs) are seen as a promising method for learning from real-world experiences [1,2]. In an LHS, health care and research are aligned to accelerate research and outcomes for patients and have the potential to develop scientific knowledge based on health information and research data by directly implementing new insights from analyses to the clinical practice [3].

For some patient populations, an LHS approach may be considered one of the most promising ways forward, for example, the group of pregnant and lactating people, who are often excluded from controlled clinical research studies and for whom there is still a poor evidence base for medication safety and efficacy. In real life, numerous medications, which are key to the health of the pregnant person, have been used safely and effectively in pregnancy with minimal risk to the fetus and pregnant person, but we do not systematically learn from these experiences [4-8]. Current information on medications used during pregnancy and lactation is fragmented and spread across different countries and data sources, including pregnancy or medicine cohorts, registries, research groups, and the pharmaceutical industry [9]. Examples of such data sources are the European system for the evaluation of safety of medication use in pregnancy in relation to risk of congenital anomalies (EUROmediCAT), the European Network of Teratology Information Services (ENTIS), and national population registries or regional cohorts. Accessing and analyzing these unique data sources in a system of continuous learning could help more effectively clarify how medications impact pregnancy outcomes.

In general, a remaining challenge of data-intensive health research, which is at the core of an LHS, has been obtaining meaningful access to data. A way to impact the field of pregnancy and lactation is through collaborations between various organizations (including public-public and public-private). These organizations, known as data access providers (DAPs), often possess or have access to vast amounts of routine (health care) data, which reflect routine health care encounters and processes, and they have valuable expertise in managing large data sets. Collaborating with private organizations can also be beneficial, as they also possess relevant data and resources. In addition, private organizations, such as medicines marketing authorization holders, require evidence on the effects of medications during pregnancy to comply with regulatory requirements and to update product information. Public-private partnerships present their own set of challenges, such as ownership, benefits and effectiveness, impact on public interest, and achieving a social license, all of which have been discussed in the literature on public-private partnerships [10,11]. In addition, frequently discussed in the context of LHS development is the challenge of establishing a sustainable collaboration capable of consistently facilitating the processes of data collection, analyses, and dissemination of research results [2,12-14].

At the same time, there is a growing expectation for these DAPs as data controllers and processors to extend their focus beyond regulatory compliance and actively safeguard the privacy and appropriate use of data. The General Data Protection Regulation (GDPR) includes various rules and principles for data controllers to ensure transparency and adherence to principles, such as fairness, purpose limitation, data minimization, accuracy, storage limitation, integrity and confidentiality, and accountability, while granting certain rights to persons whose personal data are being processed (GDPR, Articles 5 and 6) [15]. Ultimately, DAPs are viewed as moral agents who must respect ethical principles such as transparency, trustworthiness, responsibility, and community engagement [16].

To realize a sustainable and ethically responsible LHS, it is important to know whether people working for these organizations acknowledge their role and responsibility in safeguarding the responsible use of data and the dissemination of research outcomes to the public. Rising expectations with respect to DAPs’ responsibility for the ethical use of data and data ownership do not necessarily mean that each of these organizations has a dedicated governance structure to safeguard these principles or that people working for DAPs feel as if they are a moral actor in an LHS. Moreover, apart from the obvious differences in management and reward systems among DAPs [17], these organizations may also have different motivations for collaborating in an LHS. Furthermore, their perspectives on the sustainability of an LHS and their roles once the project phase concludes may also diverge.

### Objectives

In this study, we aimed to explore the views of people working for DAPs who participate in public-private partnerships to build a sustainable LHS. We were especially interested in the views of DAPs contributing to the Innovative Medicines Initiative (IMI) ConcePTION (Continuum of Evidence from Pregnancy Exposures, Reproductive Toxicology and Breastfeeding to Improve Outcomes Now) project, which aims to build an LHS for pregnant and lactating people [18]. Using a qualitative interview design, we hoped to identify, better understand, and juxtapose people’s views and interests in collaborating in an ecosystem that uses routine health data to generate new knowledge for pregnant and lactating people and their doctors. By providing insight into the views and interests of people representing DAPs in this particular LHS, this study intends to inform a governance framework for LHSs and, in turn, to help facilitate the development of a sustainable LHS in which public and private organizations collaborate. Moreover, this study aims to contribute to the ongoing discourse on moral responsibilities associated with responsible data handling and dissemination of research findings, particularly by exploring whether DAPs themselves perceive and articulate this moral responsibility.

## Methods

### Design

We conducted a qualitative study to collect the views and interests of people who work for organizations and who act as a DAP in the ConcePTION project. This qualitative interview study is a substudy of the IMI ConcePTION project (Textbox 1). IMI ConcePTION was used as the primary case study during the interviews and as the source for participation selection. The study was reported following the COREQ (Consolidated Criteria for Reporting Qualitative Research) [19]. We conducted semistructured interviews with a topic list (refer to the general topic list in Textbox 2). The topic list was based on the topic list used for another qualitative interview study, in which we asked women during preconception, pregnancy, and nursing what they thought about an LHS for pregnant and lactating women [20]. The topic list was also based on an analysis of the challenges of public-private partnerships, LHSs, and responsible data sharing [1,10,21], as well as discussions among the research team. To mitigate the potential for socially desirable responses from our respondents, it was determined that the topic of moral responsibility regarding the use of data and the dissemination of research findings would not be included in the general topic list. Instead, an opportunity for spontaneous or organic discussion of the topic was provided during the course of the interview. Moreover, it was expected to be, for example, discussed under topic 2: “expertise and dual roles.” This topic provided an opportunity for DAPs to elucidate their roles and responsibilities concerning their primary organization; their involvement in the ConcePTION consortium; and in certain instances, their clinical obligations.

**Description of the initiation, aim, and composition of the Innovative Medicines Initiative (IMI) ConcePTION (Continuum of Evidence from Pregnancy Exposures, Reproductive Toxicology and Breastfeeding to Improve Outcomes Now) project.**

In April 2019, the IMI ConcePTION project was launched, which aims to establish a trusted ecosystem that can efficiently, systematically, and in an ethically responsible manner generate and disseminate reliable evidence-based information regarding the effects of medications used during pregnancy and breastfeeding to women and their health care providers. The ConcePTION consortium consists of European public and private stakeholders, including national public health institutes, the European systems for the evaluation of safety of medication use in pregnancy in relation to risk of congenital anomalies (EUROmediCAT), the European Network of Teratology Information Services (ENTIS), research institutes, universities, and pharmaceutical companies. The ConcePTION consortium is currently a public-private partnership; however, the approach of ConcePTION to collect and learn from real-world data on the safety of medicines during pregnancy and breastfeeding is similar to what may also be called a learning health care system [6].

**General topic list used during the qualitative study to guide the interviews.**

**Topic list**

- Willingness to participate
- Expertise and dual role
- Future (after consortium agreement ends)
- Conditions for working for the ConcePTION (Continuum of Evidence from Pregnancy Exposures, Reproductive Toxicology and Breastfeeding to Improve Outcomes Now) learning health care system
- Added value

### Sample and Setting

To capture a wide range of interests and perspectives (contrast maximization), a variety of people from different types of organizations and different countries were identified. We aimed to include people working as DAPs in partnering organizations and third parties in the ConcePTION project. To be able to invite people working for different DAPs, we distinguished between private (pharmaceutical companies and private centers) and public organizations (universities, teratology information centers, public health services, and hospitals), countries, regions, collaborative partnerships, and occupations. Respondents were recruited using purposeful sampling with the help of colleagues from the ConcePTION consortium. The respondents were approached via email. Most of the interviews started with an introductory question related to the work of the respondent and the process of data collection, storage, and analysis within their organization. We then used the topic list to continue with the interview. Although the approach of ConcePTION is similar to that of an LHS, we used the terms ecosystem and network interchangeably. This is because the term ecosystem is commonly used within the consortium and is more familiar to the respondents. The interviewer (MJH) created a safe space for respondents and invited them to share their views and experiences by emphasizing (1) the privacy and confidentiality arrangements, (2) their autonomy during the interview (eg, regarding answering questions, stopping the interview, and asking for clarification), and (3) the option to review the transcript before analysis. These points were emphasized by the interviewer before seeking verbal consent. The interview allowed respondents to introduce or emphasize new issues that they considered relevant. Therefore, it is important to emphasize that the results reflect personal views and do not represent the views of the entire organization for which the respondents work.

### Data Collection

The interviews were conducted by MJH (trained qualitative researcher, female) using the topic list. The topic list was refined after 2 pilot interviews. Furthermore, according to the technique of constant comparative analysis, the interview topics evolved as the interviews progressed alongside data analysis [22]. Data were collected from November 2021 to February 2022. The interviews were conducted in English and Dutch and took place via a secure communication platform. The interviews took 33 to 60 minutes, with an average duration of 43.8 (SD 75) minutes. In 12 out of 14 interviews, there had been no previous contact between the interviewer and the respondent. In 2 out of 14 interviews, the interviewer and the respondent had contacted each other before for project-related work. During and after the interview, MJH made notes to enhance the data and to provide a clear context for data analysis. The interviews were audiotaped, transcribed verbatim, coded, and stored anonymously. Verbal consent was obtained from all the respondents. One respondent requested to read the transcript before analysis.

### Data Analysis

After transcription, we analyzed the interviews according to the thematic analysis method and by using a backward and forward approach between data collection and analysis to develop codes [22]. An initial coding list was developed based on the topic list. Subsequently, the transcripts were coded. The coding list was evaluated and adapted, and all interviews were coded using NVivo 12 software (Lumivero). To enhance the validity of our results, an intern (medical student, Bachelor of Science) also read and coded 8 randomly chosen interviews out of 14 pseudonymized interviews to check for consistency of the thematic framework and critically read the coding list. In the course of the analysis, codes were adapted, and additional codes were added to the coding list where necessary. A meaning pattern was identified across the data set, leading to the formulation of interpretative higher-order themes. The themes capture the views and interests of the DAPs regarding the ConcePTION ecosystem. The themes represent both topics that were often discussed by respondents and a variety of views that are helpful in the development of a sustainable ecosystem of continuous learning. The findings, including the coding list and formulated higher-order themes, were discussed by the complete research team (MJH, RvdG, MCJMS, and JJMvD). Thematic saturation was reached when additional data did not lead to any new emergent themes after 14 interviews [23]. Furthermore, a member check was executed during the last phase of data analysis. A draft version of the manuscript was sent to all respondents, inviting them to provide feedback and discuss the accuracy and interpretation of our results [24].

### Ethical Considerations

The research protocol, including the procedure for obtaining informed consent, was reviewed by the institutional research support office at UMC Utrecht. As no intervention was imposed on the participants, this study was exempt from ethics review under the Dutch law. All participants were provided with a letter of information and gave their verbal consent for participation and recording as required under the Dutch law that implements the GDPR (*uitvoeringswet algemene verordening gegevensbescherming*). Each participant was assigned a study ID number to protect their privacy and confidentiality. Furthermore, their names, the names of their workplace, and other names of the consortium members mentioned in the interviews were redacted by the interviewer MJH. The participants were not compensated for participating in the study.

## Results

### Overview

Of the 23 DAPs that were approached, 14 agreed to participate in the study, 4 declined, and 5 did not respond. A total of 14 semistructured interviews were conducted with 18 people involved in IMI ConcePTION. A total of 2 DAPs were represented by 2 employees of the same organization or research collaboration. The interview respondents worked in different organizations, including universities, public health centers, hospitals, teratology information centers, pharmaceutical companies, and private centers. Table 1 shows the respondents’ characteristics. We could not share all details to ensure the privacy of the respondents.

Because of the constant comparative analysis during the qualitative study, we enhanced our interview guide. During the first couple of interviews, the subject of (moral) responsibility was not (always) organically discussed. Therefore, we added to the second topic “expertise and dual roles,” the possibility of asking DAPs directly about their sense of responsibility and to whom that responsibility was directed, if relevant. We still decided to leave the answers open and not steer too much in the direction of the sense of *moral* responsibility regarding the use of health data and dissemination of research findings to avoid socially desirable answers.

On the basis of the interviews, we formulated 3 main themes characterizing the views and reflections of DAPs on the development of a knowledge-generating ecosystem for pregnant and lactating people. These themes emerged consistently across all interviews. We provide representative quotations to illustrate these themes (Table 2).

**Table 1:** List of characteristics of the respondents, categorized based on the respondent number, type of organization, whether it is a public or private organization, and the general location of the organization.

Respondent number	Type of organization	Public or private organization	General location of the organization
R01	University	Public	Southern Europe
R02	Research institute	Public	Southern Europe
R03	Pharmacoepidemiologic research institute	Public	Central Europe
R04	Research institute	Public	Northwestern Europe
R05	Hospital	Public	Central Europe
R06	University	Public	Northern Europe
R07	University	Public	Western Europe
R08	Pharmaceutical company	Private	Central Europe
R09	Public health service	Public	Middle East
R10	Pharmaceutical company	Private	Western Europe
R11	University	Public	Northwestern Europe
R12	Hospital	Public	Northwestern Europe
R13	Health center	Private	Middle East
R14	University	Public	Northwestern Europe

**Table 2:** Representative quotations (Q) from the respondents (R) used to illustrate the identified themes.

Themes and quotation number			Quotation and respondent number
**Theme 1: opportunity and responsibility**			
	Q1	“It was another opportunity for us to exchange data on a wider basis. ...share with one another might be an interesting experience.” [R09]	
	Q2	“The first thing to remember, is that we want to be important. We want to continue being bold. Because at the end, it’s big; ConcePTION. It has a lot of power. We want to be there. Not for, only for some type, scientific purposes. But the main one is, to include our data.” [R02]	
	Q3	“Think it’s two things. One is we feel the obligation, because we have a large database, so it’s a moral obligation I think—or we think. And the other one is also because we like working in this team.” [R03]	
	Q4	“I’m excited to be in this field, because you can help people improve their health whether it’s women or children, doing this study, or in other types of study we do. I’m not sure I’d use the word responsible in that context, but definitely it’s a motivating factor.” [R14]	
	Q5	“Then we would have some safeguards that we are the ones who say ‘Yes, this data can be used,’ or the results. We have obligations to the data providers; we need that these are full in. So the problem is if we have like one day to review the results and then something is published, we will kind of have problems with our obligations.” [R03]	
**Theme 2: conditions for participation and commitment**			
	Q6	“To be sure that at least we have one [person] working on this. And that it is a very stable income. Because otherwise we are looking for the calls [tenders] and running for them. And yeah, it takes a lot of time, and when we spend time on this, we don’t spend time on thinking about the research we’re performing.” [R07]	
	Q7	“We are a research institute, and we get evaluated every seven years, and we are measured on publications mostly. So, research is a value for us and publications is important for us, and especially also first and last authorships. So we need to focus our resources on getting some publications.” [R03]	
	Q8	“There needs to be some rules, an agreement about our participation and how much pharma can affect the processes and how much pharma can receive from this and every package actually, so it should be in some agreement written down.” [R13]	
	Q9	“What I would want is to have more time to discuss things like double programming and also to decide like decisions implicitly made.” [R03]	
**Theme 3: challenges for a knowledge-generating ecosystem**			
	Q10	“[In] the end, you’re going to need a person who understands the data and to analyze [the data] how is the meaning of the data? Because if you are, at the end, just a numbers situation. You are not thinking about this biological play. Classical or not. Or if it makes sense with your kind of population.” [R02]	
	Q11	“In many countries that are strict data privacy rules and when for a given observation, there are like less than four observations, the results are masked. ...that means that I cannot use the data when combining data from several studies. So one thing that I think would be beneficial is to see if there would be data privacy rules that would be lifted for pregnancy studies.” [R08]	
	Q12	“So, but it’s a big assumption. Because academia is involved, you know,..., taking care of [the governance; the data privacy]. And ...they will handle the trust part. I trust them or [when academia] are taking the lead in this project, I’m like: ‘okay I think they will take care of everything.’ ...They [academic partners] are extra careful, and that extra carefulness is making collaborating complex and difficult.” [R10]	
	Q13	“But here one of the biggest questions is the sustainability. So how this platform will be, I’m saying platform and it’s not the exact quote, but how this platform will be sustained after ConcePTION.” [R08]	

### Theme 1: Opportunity and Responsibility

Most respondents wanted to contribute to the ConcePTION project because they viewed the project as an *opportunity* to (1) contribute to the goal of creating knowledge on the safety of medication used during pregnancy and lactation; (2) look at medication safety and birth defects in a larger context (European wide); (3) collaborate and share experiences with other registries, databases, and the like (quotation 1); (4) stimulate scientific research; (5) learn from others and their registries; and (6) showcase their databases and share expertise (quotation 2).

Respondents also emphasized the need to use real-world data. Some respondents mentioned that they feel it is their *responsibility*, or as 1 respondent expressed, *moral obligation* to contribute because of the database or resources they have access to. They felt that they, with their organization, were in a position to contribute to something important, and therefore, they must (quotation 3). Some have been working for a very long time on this specific topic and have already contributed greatly to solutions to close the knowledge gap regarding medication safety in pregnancy and lactation. Only a few mentioned that they felt responsible for helping these groups of people; others saw the lack of knowledge more as a motivation to contribute to the ConcePTION ecosystem (quotation 4).

Besides articulating a responsibility toward pregnant and lactating people, their offspring, and their doctors, the respondents of the private industry also explained that they need to generate knowledge because it is a requirement from the European Medicines Agency and Food and Drug Administration. As they are required to research medication safety among pregnant people, this was considered to be another type of obligation and, with that, a different type of willingness to participate.

A few respondents also expressed feeling a responsibility for enabling research and the quality of the data analyses, and because of that, they wanted to be involved in the decision-making regarding the development and testing of analytical scripts within the research ecosystem.

Finally, 1 respondent also mentioned their responsibility and obligations toward other *data providers*. Some organizations receive data from other organizations, such as health insurance providers. Because of these obligations, they wanted to remain in control of some of the review processes in terms of data programming and analyses (quotation 5). However, challenges in this regard were also discussed stemming from time and financial constraints as well as short research deadlines. None of the respondents discussed their role as data controllers, which involves the responsibility to determine the purpose and manner in which personal data are processed.

### Theme 2: Conditions for Participation and Commitment

Respondents explained that their willingness to collaborate within the ConcePTION LHS depends on certain conditions that need to be in place.

#### Resources and Support

In all interviews, financial resources were discussed as an important condition. Interestingly, financial resources were mentioned as important for reasons beyond the immediate need to cover resource costs associated with participation in a project. Financial resources were discussed in the following ways: (1) as a stable flow of income, preferably contracted for an extended period and covering all the planned activities, and (2) as a source of funding. A stable flow of income is beneficial for attracting and training more employees in this area of work and will help with distributing tasks and becoming more specialized and efficient in the field of pharmacoepidemiology. Agreements on financial support are also necessary for planning and being less dependent on other sources to keep “the system running” (ie, tendering; quotation 6). Regarding sources of funding, some respondents specifically stated that they cannot receive funding from the private industry. They believe that because they are independent (public) institutions, there would be a conflict of interest.

Other respondents mentioned that besides financial resources, they also need IT and computational resources to perform the actual analyses and to ensure that they can keep up with the heavy computational work, which is necessary for sustaining the data analyses.

Some respondents mentioned that they are not used to writing certain types of protocols or experience challenges when receiving ethics approval for studies. Some respondents suggested that ConcePTION could benefit from having a permanent staff to provide support and address questions about timelines, deadlines, funding, ethics, and events.

#### Scientific Output and Motivation

The importance of scientific output was emphasized during the interviews. Some respondents worked in academic institutions whose aim was to produce scientific publications (quotation 7). Therefore, their willingness to participate in an ecosystem is also affected by whether they get to perform and design studies within the ConcePTION LHS and publish the results in scientific journals. Some respondents also emphasized the need to ask more scientific questions and implement more scientific methods within the network. They mentioned that working within the ConcePTION ecosystem should be different from tendering for projects from pharmaceutical companies. Finally, respondents also wanted to feel motivated to commit to the ConcePTION ecosystem. According to them, motivation is stimulated in different ways, but most importantly, by scientific interest in the project, autonomy regarding work, respect for expertise, and good working relationships. A few respondents also emphasized the importance of offering valuable and easily accessible knowledge to pregnant and lactating people as well as health care providers as a prerequisite for contributing to the ecosystem. They felt that generating valuable information for these stakeholders is the most important goal of an ecosystem such as ConcePTION.

#### Safeguards

Safeguards were also mentioned as a condition for working for the ConcePTION ecosystem. A few respondents were hesitant regarding the role of the pharmaceutical industry in the processes of formulating research questions, cowriting protocols, and analyzing results (quotation 8). According to them, industry involvement could conflict with the primary goal of the research, or they considered it challenging to align the goals of private and public industries. Other respondents, who worked for pharmaceutical companies, regretted this view and argued that collaboration is very much needed and possible because of independently determined regulations that govern both public and private organization research into the effects of medicines. They stressed that trust and open-mindedness toward each other are important for a good collaboration.

Another safeguard mentioned by some respondents was related to transparency. They argued that in a large network and with a developing ecosystem, it is important to be able to track every step and decision made regarding techniques and methods. One respondent explained how several decisions are made in the process of data analyses, which can influence the quality and value of the results (quotation 9). A few respondents also mentioned that to safeguard the quality of data analyses, especially in the developmental phase of the ecosystem, decisions about technical aspects such as programming and writing scripts for analyses need to be transparent for all DAPs. In this way, DAPs can perform their own quality checks, if desired, and provide valuable feedback.

### Theme 3: Challenges for a Knowledge-Generating Ecosystem

When asked about their perspective on the development of a knowledge-generating ecosystem, respondents talked about the challenges they have experienced thus far and which, according to them, are relevant when building the ecosystem.

#### Data (Is Not Information)

Some respondents explained that there were challenges in harmonizing the databases and executing studies because of the heterogeneity of the data across all databases. Some respondents also mentioned that it may be challenging to generate reliable information based on such heterogenic data, databases, and IT systems. Most importantly, data are not (yet) information or knowledge. To overcome this challenge, respondents discussed 3 types of solutions. First, to be able to interpret data and develop valuable information, many respondents emphasized the need to involve experts who know the data and the real-life health care context of the persons whose personal data are being processed and data points represented in the different data sets (quotation 10). Second, respondents mentioned the need for security and quality assessments to ensure that analytic scripts fit the data and are run correctly at every organization. Third, a few respondents preferred to work in small teams so that they could exchange experiences with scripts, data analyses, and research questions. According to them, working in small teams creates a better overview of the possibilities and limitations of data.

#### Governance

Some respondents experienced challenges owing to governance procedures. On the one hand, it was mentioned that these procedures are challenging because countries have different data privacy rules, which sometimes complicate the ability to perform observational studies (quotation 11). On the other hand, it was mentioned that these procedures are challenging because their own company or organization restricts certain (research) activities. Some respondents argued that in academia, people exert extreme caution regarding governance, which creates an additional barrier to collecting, sharing, and analyzing data. One respondent assumed that the involvement of academic institutions in the consortium implied that matters such as data handling, privacy and confidentiality, and trust were adequately addressed. However, according to the respondent, this also led to an increase in bureaucratic steps, making collaboration more intricate and challenging (quotation 12). Furthermore, respondents agreed that having fragmented governance procedures led to slow processes and unfulfilled opportunities. According to these respondents, a clear overview of what can be done with the data could be of great help.

Concerning governance, some respondents discussed the need for trust between all collaborators, especially regarding the aim of the ecosystem and methods used within the ecosystem. It was also mentioned that people need to trust the decisions made by people taking a more leading role in the ecosystem and that trust between the public and private participants is necessary to ensure that robust knowledge is going to be generated transparently within the ecosystem. Finally, many respondents emphasized the need for a good sustainability model for the ConcePTION LHS (quotation 13).

## Discussion

### Principal Findings

The results of our analysis indicate that respondents felt responsible to participate in an LHS for pregnant and lactating people. Although respondents emphasized the professional opportunities that come with participating in a large public-private partnership, many respondents collaborated because they wanted to help develop an ecosystem that can transform real-world data into new knowledge on medication safety and efficacy.

### Moral Responsibility

From our interviews, it seems that people mainly reflect upon their views and responsibilities from the perspective of their professional role as a data analyst or pharmacoepidemiologist. As a result, most answers were linked to the more technical side of realizing a system in which real-world data can be used, together with a sense of moral responsibility toward the quality of their data, databases, and data analyses (under theme 1 and as mentioned in quotation 5). On the one hand, technological responses are not surprising because of the expertise of our respondents. On the other hand, our respondents work at the core of data processing and analysis, which means that their role is also to handle the data ethically. Some respondents mentioned that they assume that compliance with rules and regulations is being taken care of by other departments of their organization or other people within the LHS, and therefore, they did not worry so much about the ethical handling of data. However, compliance with rules and regulations is a narrow understanding of handling data ethically because it often solely refers to protecting the privacy and confidentiality of persons whose personal data are being processed—an aspect extensively discussed in the interviews and sometimes perceived as a complicating factor for research. Although many respondents viewed contributing to ConcePTION as an opportunity to generate new information for pregnant and lactating people, there appears to be a lack of widespread moral responsibility toward handling data from the perspective of pregnant and lactating people. Some respondents also considered pregnant and lactating people themselves to be disconnected from the work they are responsible for. However, during the member check, some respondents expressed that they did not feel accurately represented in the portrayal of their views on this topic. For them, it was important to recognize that they feel responsible for contributing to the ConcePTION project [25].

### Trust and Transparency

Interestingly, trust and transparency were discussed as important aspects of the relationship between the participating organizations. Respondents explained that trust and open-mindedness are important conditions for working toward a common data model and getting everyone to share the same vision for the LHS. In the literature on public-private partnerships, big data research, and data-intensive research in health care, trust is also often mentioned as a crucial principle for effective collaboration [10,26,27]. During the interviews, there was hesitancy among respondents about the prospects of public-private collaboration. Some respondents mentioned that they believe they are officially constrained by their institution to closely collaborate with the pharmaceutical industry or cannot share any data (pseudonymized or not) with the pharmaceutical industry. This constraint challenges the effectiveness of the collaboration and, as a result, might complicate the development of a sustainable LHS as a public-private partnership. Interestingly, the ConcePTION project currently operates as a consortium under a consortium agreement, making reference to the European Network of Centers for Pharmacoepidemiology and Pharmacovigilance code of conduct (2010) [28]. The European Network of Centers for Pharmacoepidemiology and Pharmacovigilance code of conduct aims to maximize transparency and promote scientific independence. Furthermore, a consortium agreement typically addresses the issues of a conflict of interest by making agreements on ownership and intellectual property, obligations and rights of the participating parties, and third-party agreements. It seems that although many of the concerns of our respondents are addressed in the consortium agreement, they are not aware of these arrangements or they still experience dilemmas regarding the collaboration and their own interests, which can lead to a continued lack of trust between the public and private industries. It might be worthwhile to close this gap between the consortium agreements and the experiences of collaborators by ensuring that everyone understands the consortium structure. In the literature on large research consortia, it has been argued that transparency is important for realizing an appropriate governance framework for these types of complex collaborations. Here, transparency refers to the accessibility and visibility of the governance structures. For example, within a consortium, good governance requires that those internal or external to the project know what governance structures and procedures are in place, what mechanisms for legitimate decision-making have been adopted, and where the authority and responsibility for different types of actions are located in the consortium [17]. Our interviews underline the importance of transparency in the context of governance of an LHS with public and private organizations. One solution is the installation of a separate independent body, especially when the contractual agreement of the consortium has ended. Some scholars have suggested a Data Access Committee that can help protect persons whose personal data are being processed from foreseeable harm, stimulate social value, and mandate clear lines of accountability, terms of reference, and membership [29].

### Public Trust

The above-described perceptions of trust are of course important; however, both the literature and our previous interview study with women during preconception, pregnancy, and nursing show that public trust is also of crucial importance for the development of an LHS [20]. In the literature, it is emphasized that it is important to meet the public expectations for transparency when developing an LHS, which in turn will strengthen or maintain trust in not only the LHS but also the institutions working within the LHS [26]. People anticipate that their voluntary contribution of data will be used to enhance the care for others and they expect that their good faith will not be taken advantage of. Therefore, much depends on the extent to which uses of personal data are seen as serving the public interest and conducted by those with a public interest orientation. It is of great importance that in an LHS, public interest is considered to realize transparency, increase responsibility, and earn the trust of the public. Interestingly, some of our respondents seem to expect that others in their organization are taking care of these principles that are important for public trust or are, again, not fully aware of the governance and arrangements within the organization or the collaboration.

### Future of an LHS for Pregnant and Lactating People

Many respondents viewed the ability to conduct scientific research within a broader context as a crucial opportunity. Engaging with a diverse range of organizations can not only enhance the quality of data analyses but also improve the integrity of individual databases. Although research is essential in a knowledge-generating ecosystem, the implementation of research within the health care system is equally important. Respondents affiliated with academic institutions emphasized the significance of publishing new findings in scientific journals, as this is a key aspect of their professional responsibilities. In an LHS, it is imperative to move beyond the conventional practice of publishing primarily in scientific journals and instead prioritize the ethical integration of learning within the delivery of care [30]. This approach would allow for the continuous improvement of care through the application of new insights, while also ensuring the proper management of data. Pharmaceutical companies have already applied this method to a certain extent by generating evidence and translating findings onto product labels and educational materials for health care providers. Perhaps, the dissemination of new insights is an area in which these parties should work together and learn from each other. As LHSs mature, it is crucial that all stakeholders recognize and embrace the system’s necessity and value, extending beyond the project phase to include patients, physicians, scientists, institutional boards, pharmaceutical companies, governments, and other relevant parties.

### Limitations

Our study had several limitations. First, we have tried to purposefully include both public and private industry partners; however, we have received more responses from people working in public organizations. Thus, we were not able to include people working in the eastern part of Europe, which challenges the generalizability of our findings, as Eastern European organizations might reflect a different culture and attitude toward an LHS. Second, although we wanted to avoid socially desirable responses, the topic of moral responsibility regarding data handling was not always organically discussed during the interviews. To address this topic, the interviewer directly asked some respondents about their sense of responsibility for specific aspects of their work. Openly discussing the topic could have influenced the initial position of the respondent. We would also like to emphasize that we spoke to individuals who represent their organization in the context of the consortium; however, they do not represent the views of their organizations. Therefore, their views were subjective and might differ from those of other people working in the same organization. It would be interesting to understand the views of DAPs outside the context of pregnancy. As mentioned in the *Introduction* section, in many areas of health care, LHSs are seen as a promising way to learn from real-world data. To establish a successful LHS, more research is needed on the perspectives of the stakeholders involved.

### Conclusions

To conclude, people working for DAPs have different reasons for contributing to a project such as IMI ConcePTION, which aims to build an LHS for pregnant and lactating people. The most common motivation was opportunity. The opportunities included creating knowledge on medication safety during pregnancy, examining medication safety in the European context, collaborating with and learning from other experts, stimulating scientific research, presenting their database, and securing financial support. Although many respondents expressed a responsibility to enable real-world data analyses, their focus was primarily on their work and contribution to the project rather than safeguarding ethical data handling from the perspective of pregnant and lactating people. The results of our interviews underline the importance of a transparent governance structure that addresses decision-making processes, authority, responsibility, and accountability. Trust is crucial for the success and sustainability of a public-private LHS, relying on the relationship between DAPs and public trust. For an LHS, it is essential that all relevant stakeholders recognize and embrace the need for and added value of the system itself.

## Abbreviations

ConcePTION: Continuum of Evidence from Pregnancy Exposures, Reproductive Toxicology and Breastfeeding to Improve Outcomes Now
COREQ: Consolidated Criteria for Reporting Qualitative Research
DAP: data access provider
ENTIS: European Network of Teratology Information Services
GDPR: General Data Protection Regulation
IMI: Innovative Medicines Initiative
LHS: learning health care system

## Data Availability

The data sets generated and analyzed during this study are not publicly available because individual privacy could be compromised. In addition, no permission was obtained from the participants for public availability. The data set is available from the corresponding author (MJH) upon reasonable request.
